# Does functional fitness decline in accordance with our expectation? – a pilot study in healthy female

**DOI:** 10.1186/s13102-015-0012-y

**Published:** 2015-07-10

**Authors:** Yin-Shin Lee, Li-Ying Chang, Wei-Hsuan Chung, Tsung-Ching Lin, Tzyy-Yuang Shiang

**Affiliations:** 1 Department of Physical Education, National Taiwan Normal University, Taipei, TW Taiwan; 2 Department of Athletic Performance, National Taiwan Normal University, Taipei, TW Taiwan; 3 Department of Physical Medicine and Rehabilitation, Far Eastern Memorial Hospital, Taipei, TW Taiwan

**Keywords:** Elderly people, Lower extremity muscle strength, Exercise activity

## Abstract

**Background:**

Aging may cause various functional abilities gradually deteriorate. With changes in social forms, the trend of functional fitness decline will change accordingly. Therefore, this study endeavored to identify the trends in functional fitness decline by comparing the differences in the functional fitness of females in various age groups.

**Methods:**

Thirty six healthy females were divided into 3 age groups: young healthy females (20 to 30 y); middle-age (45 to 55 y); and older (65 to 75 y). Functional fitness test battery included flexibility, muscle strength/endurance, aerobic endurance, balance and agility.

**Results:**

The performance in the elderly group was significantly worse (*P* < .05) in all the tests, whereas the muscle strength and endurance, as well as aerobic endurance for the middle-age group showed significantly lower than young groups (*P* < .05).

**Conclusions:**

The reduction in lower extremity muscle strength occurs in the middle-age group. We recommend that middle-age women be conscious of the reduction in their lower extremity muscle strength and conduct advanced preparations for future aging.

## Background

Aging is the accumulation of changes in a person’s bodily structures and functions over time, and is a common, irreversible, and continuous process. Furthermore, various bodily abilities gradually deteriorate with time. Specifically, degeneration and deterioration of muscles is a crucial factor influencing normal functions in daily life [1, 2]. Previous studies have indicated that after 40 years of age, muscle strength decreases annually by an average of 1 %; after 60 years of age, the extent of reduction becomes more substantial; between 60 and 70 years of age, muscle strength decreases by approximately 15 %; and, compared to young people, the muscle mass of elderly people above 65 years of age and above 80 years of age decreases by approximately 9 % to 18 %, and 30 %, respectively [3]. Because muscle mass decreases, muscle strength declines; thus, bodily functions deteriorate, resulting in action impairment and a dramatic increase in the risk of falling and mortality rates [4]. Currently, with changes in social forms or patterns, middle-age women are generally struggling with the dual responsibilities of family and work; therefore, compared to other age groups, these women have less time for exercise activities [5]. Consequently, the fitness changes in middle-aged are extremely important. Most of studies have mainly adopted elderly people as primary research participants or have generally compared the differences between young and elderly people [6, 7]. However, it is also important to compare the differences among young, middle-age, and elderly people to observe the trends by which abilities significantly deteriorate [8].

Both muscle mass and muscle function decrease as people age, which ultimately causes muscle strength to decline. This phenomenon is known as sarcopenia [9, 10]. In severe cases, overall health declines, resulting in poorer quality of life and even disabilities. Sarcopenia poses serious health threats to elderly people, with the most obvious symptoms being a reduction in muscle density and increase in adipose tissue between the muscles of middle-aged and elderly females [1, 3, 11]. Moreover, inadequate exercise or bodily activities and acceleration of loss in muscle strength creates a vicious cycle that leads to massive loss of bone density, rapid reduction in muscle strength, and a significant increase in the probability of contracting chronic diseases such as cardiovascular disease and diabetes over the long term [12]. In particular, post-menopausal women should be aware of whether the above phenomena occur in their personal lives.

Previous studies have developed a serious test of functional fitness to assess the physiological parameters which presenting the physical ability of elder people [13, 14]. Aging necessarily results in the deterioration of bodily functions (eg, muscle strength, endurance, Aerobic endurance, flexibility, et al.) that influence the execution of various activities in daily life, and the performance of aging people regarding functional fitness is undoubtedly poorer than that of young people. However, functional fitness also deteriorates or changes as a result of a lack of activity. Numerous studies have confirmed that sufficient exercise or bodily activities assist elderly people in maintaining their functional fitness and keeping healthy [15–19]. Nevertheless, understanding when the critical period during which forms of fitness deteriorate occurs is crucial. We should also focus on comparisons among young, middle-age, and elderly people. Using the concept of prevention as a basis, researchers should understand the actual condition of fitness deterioration and when various forms of fitness begin to show significant deterioration to provide early intervention for enhancing exercise or bodily activities. This can consequently improve the fitness of elderly people. Therefore, this study compares the differences of the functional fitness among young, middle-age, and elderly people to identify the trends of decline for various forms of fitness, and subsequently adopts the results as a basis for determining aging in various age groups and to provide recommendations and references regarding exercise prescriptions for middle age and elderly people. We hypothesized that the functional fitness will deteriorate gradually with aging, but deteriorate sharply in elderly people.

## Methods

### Participants and recruitment

We recruited 90 healthy and functional independent female adults (20 to 75 y of age). Then we selected 36 participants with similar body weight and height who were divided into 3 groups: young healthy females (YHF, 21.42 ± 1.62 y of age, height: 161.43 ± 6.24 cm, weight: 56.01 ± 5.06 kg, BMI: 21.51 ± 1.74, collage student), middle-age healthy females (MHF, 52.42 ± 0.69 y of age, height: 156.33 ± 4.60 cm, weight: 57.52 ± 8.12 kg, BMI: 22.87 ± 1.70, household), and older healthy females (OHF, 71.08 ± 3.37 y of age, height: 156.42 ± 4.74 cm, weight: 55.23 ± 6.26 kg, BMI: 22.03 ± 1.94, free-living). Each group included 12 participants. None of the participants have the experience of resistance weight training and aerobic fitness training. Participants were screened using the following exclusion criteria: presence of hip fracture, uncontrolled hypertension (>150/90 mm Hg), stroke, myocardial infarction, nephropathy, or other rare congenital diseases [20]. Participants who were injured in the previous 6 months and were required to undergo medical treatment as a result were also excluded. This study was approved by the institutional review board of Taipei Medical University, and all participants were provided with information about this study and their signed consent was required.

### Functional fitness tests

In this study, two assessors were recruited from the department of athletic performance of a local university who trained to execute an assessment, and the first participant of young group was repeated twice to make sure we get consistent result (The interrater reliability was .99 between assessors). Seven tests were chosen to assess the functional fitness of the 3 groups. Those tests have been proved to have acceptable validity and reliability following the appropriate scientific standards [13, 14, 21].

1. Flexibility: Chair sit-and-reach test
  This test was used to measure lower body flexibility by evaluating the distance between extended hands and toes at full extension. Participants were asked to sit at the front half of a chair, with one leg (ie, the dominant leg) extended as straight as possible and the ankle flexed at approximately 90° with only the heel placed on the floor. The other leg was bent with the foot flat on the floor. With outstretched arms and overlapped hands, participants slowly bent their hip joint forward and touched their toes. If the middle fingers touched the toes, the score was 0. If not, the score was negative.
2. Lower extremity performance:
  A. Extensor muscle strength: Leg press test
    Before the test, participants were asked to stand on the leg press device (TTM-QM; Accuratus, TW) with their feet split as wide as their shoulders. Then flexed their knee at 90° [22], gripped the handle with both hands and kept their arms and back (torso) straight with their eyes facing forward. When the signal start was provided, participants were required to extend their leg as far as possible for 5 s. This test was performed only once.
  B. Muscular endurance: 30-s chair-stand test
    Before the test, participants were asked to sit on the front of the chair with their hands across their chest. Next, they performed standing up and sitting down for 30 s. The stand-sit repetitions were recorded as the lower extremity muscular endurance score. This test was performed only once.
3. Upper extremity muscle strength: Grasp test
  Before the test, a grip device (TTM-YD; Accuratus, TW) was adjusted to fit each participant’s hand. Participants were asked to sit on the chair and device was not allowed to touch their body; in addition, their arms had to be straight and steady. The test was repeated twice, and the better of the 2 scores was recorded.
4. Aerobic endurance: 2-min step test
  Before the test, the knee-step height was set to fit each participant, which was even with the midpoint between the kneecap and anterior superior iliac spine. When the test started, participants were required to march in place as much as possible within 2-min. We only recorded the number of times when both knees had reached or surpassed the set height as the aerobic endurance score. This test was performed only once.
5. Balance: Open-eye stand-on-single-leg test
  Participants stood statically on both feet in the beginning of the test, then raised one leg. The score for the test was set as the time before the participants lost their balance (eg, the free leg touched the ground) or reach 60 s. The test was repeated twice, and the better of the 2 scores was recorded.
6. Agility: 8-foot up-and-go test
  The distance between the chair and the return point was 2.44 m. Participants were asked to stand up from the chair and walk around the return point then sit again as fast as possible. The start position was normalized by placing the knees at a 90° flexion (the chair height and foot placement were adjusted), and placing the hands beside the body. The agility score was recorded as the time it took the participants to complete the movement from the moment they left the chair (where the sensor pad was set) until they returned to it. The test was repeated twice, and the better score was recorded.
  Procedure of functional fitness test: Each participant initiated a warm-up exercise at the beginning of the test. Then did height/body weight measurement, grasp, flexibility, leg press and stand tests (30-s chair-stand). After 5-min break, balance and agility tests were performed. After another 5-min break, 2-min step test was performed.

### Statistical analysis

SPSS for Windows (version 18.0) was used to analyze the data. Kolmogorov-Smirnov test was used to check variables normally distribution. An independent one-way ANOVA was used to compare the differences in functional fitness among the different groups. Tukey post hoc tests were used to identify the statistically significant mean differences. The significant level was set at α = .05.

## Results

The participant demographic and mean score of seven tests were shown in Tables 1 and 2, respectively. The results showed that BMI (F = 2.014, *P* = .150, η2 = .109, β = .386) and flexibility (F = .088, *P* = .916, η2 = .005, β = .062) were not significantly different among the 3 groups. Regarding the lower extremity performance, the YHF performed significantly better than the MHF and the OHF in extension muscle strength (F = 13.012, *P* < .001, η2 = .448, β = .995) and muscle endurance (F = 21.002, *P* < .001, η2 = .560, β = .999).

**Table 1:** Participant demographic (N = 36)

Item	n	%
Age (years)		
20–30	12	33.3
40–50	1	2.8
50–60	11	30.6
60–70	4	11.1
70-	8	22.2
Exercise frequency		
Less than once/week	5	13.8
Once/week	1	2.8
Twice/week	4	11.1
Thrice/week	2	5.6
Fourth or more/week	24	66.7
Exercise habit		
Yes	31	86.1
No	5	13.9

**Table 2:** The mean score of functional fitness test

	YHF	MHF	OHF
BMI	21.51 ± 1.74	22.87 ± 1.70	22.03 ± 1.94
Chair sit-and-reach (cm)	11.91 ± 9.13	12.06 ± 7.70	13.13 ± 6.10
Leg press (kg)	79.86 ± 12.71^ab^	61.96 ± 10.33	52.13 ± 15.82
30-s chair-stand (times)	25.00 ± 5.70^ab^	16.17 ± 3.88	14.25 ± 2.96
Grasp (kg)	28.22 ± 5.57^b^	25.03 ± 3.70	21.85 ± 2.64
2-min step (times)	146.67 ± 22.50^ab^	124.58 ± 16.65	105.42 ± 25.55
Open-eye stand on-single-leg (s)	60.00 ± 0.00^b^	54.75 ± 11.60	30.50 ± 24.12^a^
8-foot up-and-go (s)	7.21 ± 0.60^b^	7.80 ± 1.04	9.94 ± 1.46^a^

^a^significantly different with MHF

^b^significantly different with OHF

The grasp strength of the YHF was significantly greater than that of the OHF (F = 5.718, P = .007, η2 = .257, β = .832). The aerobic endurance performance of the YHF was significantly better than that of the MHF and OHF (F = 10.676, *P* < .001, η2 = .393, β = .983). The single leg stand time of the OHF was significantly shorter than that of the YHF and MHF (F = 12.446, *P* < .001, η2 = .430, β = .993). The 8-foot up-and-go test time of the OHF was significantly longer than that of the YHF and MHF (F = 20.896, *P* < .001, η2 = .559, β = .999).

## Discussion

The objective of testing functional fitness is to understand the fitness performance of elderly people in their daily lives. The level of functional fitness is closely correlated with health [21, 23]. In particular, lower extremity muscle strength is a crucial factor that influences performance regarding other abilities (eg, dynamic/static balance and agility) and affects the ability of elderly people to live independently [17, 22, 24, 25]. In this study, the performance in all items (except BMI and flexibility) decreased with age. The absence of significant differences of BMI and flexibility between the 3 groups was probably caused by the small sample size. In addition, this finding corresponded with previous studies, which asserted that flexibility is primarily influenced by gender differences and is not significantly correlated with age [26, 27].

Other items, specifically lower extremity muscle strength, muscular endurance, and aerobic endurance, noticeably deteriorated in middle-age people. Thus, reduction in lower extremity muscle strength does not demonstrate an even or constant decrease, and began earlier than original anticipation. This phenomenon may also relate to the menopause status. It is a critical period of life associated bone loss, physical incapacity to female [28]. Moreover, most of previous researches frequently focus on the differences between young and elderly people, and it is also important to incorporate middle-age people in studies [8, 20, 29, 30]. In the present study, the results indicated that various abilities had already begun deteriorating in middle-age people, and no significant differences in these abilities were identified between middle-age and elderly people. We were only able to identify a noticeable decrease in grasp, balance, and agility at old age. Despite similar performance regarding lower extremity muscles for the elderly and middle-age groups, the balance and agility of the elderly group were comparatively poor. In addition to showing that the risk of falling for elderly people is considerably higher than that for middle-age people [31]. The results also demonstrated the complexity of the relationship between aging and the reduction or deterioration in various abilities (eg, lower extremity muscle strength, balance, and agility). Furthermore, the decline in a single function (eg, lower extremity muscle strength) may cause the loss of various abilities necessary to live or increase the risk of falling, and consequently affect the ability to live independently and influence quality of life.

According to the results obtained for the middle-age group, the performance of decrease in lower extremity muscle strength/endurance was at an identical performance to that for the elderly group. Nevertheless, the performance of the middle-age group regarding other functional fitness items (eg, balance and agility) was not worse than young group, indicating that lower extremity muscle strength/endurance is simply one among many factors influencing functional fitness decline. A previous study has indicated that the degree of body sway during walking and muscle nerve control is positively correlated with performance in lower extremity muscle strength [32]. This finding suggests that a body’s dynamic balance ability is influenced by lower extremity muscle strength and is a crucial factor that prevents falling. Because stepping and walking entail the support of a single leg movement, aging causes reductions in lateral postural balance and subsequently results in the inability to maintain frontal plane balance [1, 33]. Therefore, middle-age women should be conscious of performance decrease in lower extremity muscle strength to prepare for the influences induced by future aging. In addition, the aerobic endurance performance for the middle-age group significantly decreased partially because the deterioration in thigh muscle performance (muscle strength/endurance) caused performance in the 2-min step test to decrease [34, 35]. Furthermore, middle-age women may be frequently engaged in family responsibilities and work; hence, they are deprived of the opportunity to exercise. Consequently, their muscle strength and aerobic endurance decreases.

Although performance of the elderly and middle-age groups for lower extremity muscle strength/endurance were similar, the balance and agility performance of the elderly group were significantly poorer compared to those of the middle-age group. This result indicates that sensory integration, including proprioception, vestibular perception, and visual perception, all deteriorated noticeably [36, 37]. Previous research has contended that during the transition from double leg support to single leg support, the location of the center of mass decreases rapidly and shifts to the supporting leg, showing that elderly people require substantial hip joint and spinal strength to maintain body balance [38]. Previous studies have verified that bodily activities or resistance training reduce the occurrence of frailty and provide positive enhancements for the health of elderly people [39–44]. However, Peterson, et al. indicated that elderly people who are involved in activities of daily living only, such as gardening and non-exercise walking, have poorer functional performances (eg, gait speed, chair stand, and duration of long-distance walks) and a higher probability of becoming disabled than those who undergo exercise activities [17]. In other words, the activities in people’s daily lives are not sufficiently intense to reduce the progression of frailty. Thus, the intervention of bodily activities involving more active strategies or intense exercises is necessary to prevent the occurrence of disabilities.

Regarding tests for upper and lower extremity muscle strength, the performance in grasp decreased slower than performance concerning lower extremity muscle strength [45]. Though we didn’t check the lifestyle of our participants in this study. This phenomenon may be associated with the sedentary lifestyle that people currently lead, particularly that for modern people who have more opportunities to employ their upper extremities because of work. Consequently, upper extremity muscle strength decreases less rapidly than lower extremity muscle strength. In addition, the grasp test is a simple measurement method that evaluates muscle strength and functions. It is a highly predictive indicator for determining the mortality and morbidity rate of elderly people or disabled elderly people [46, 47]. The results of this study showed that the grasp performance of the elderly group was significantly poor compared to that of the young group. However, no differences were found between the elderly and middle-age group as well as the middle-age and young group. In present study, the deterioration of the upper extremities, in contrast to the lower extremities, is comparatively slow and subtle. Nevertheless, all people, especially elderly and middle-age people who do not work, should undertake additional exercise to reduce the rate of upper extremity deterioration.

The limitation of this study was the small size of the participant group. We only recruited healthy females with the average body build. The results may need to be replicated in larger size of participant group, different gender, or different health condition participant. The finding might differ from present study. However, in present study, we have obtained some crucial information indicating that the reduction in lower extremity muscle strength was faster as our expected.

## Conclusion

Integrating the above results, reduction in lower extremity muscle strength occurs in the middle-age group. This phenomenon is crucial and warrants further attention. Therefore, middle-age women should be cautious of the reduction in their lower extremity muscle strength. Both middle-age and elderly women should actively participate in exercise activities and, if possible, resistance training, to specifically strengthen and enhance their lower extremity muscles and conduct advanced preparation for future aging. Furthermore, to reduce the progression of balance and agility deterioration, decrease the risk of falling, and facilitate and maintain other functional fitness items and the ability to live independently.
